# Unsupervised analysis of whole transcriptome data from human pluripotent stem cells cardiac differentiation

**DOI:** 10.1038/s41598-024-52970-z

**Published:** 2024-02-07

**Authors:** Sofia P. Agostinho, Mariana A. Branco, Diogo E. S. Nogueira, Maria Margarida Diogo, Joaquim M. S. Cabral, Ana L. N. Fred, Carlos A. V. Rodrigues

**Affiliations:** 1 Department of Bioengineering, Instituto Superior Técnico, Universidade de Lisboa, Av. Rovisco Pais, 1049-001 Lisbon, Portugal; 2 iBB—Institute for Bioengineering and Biosciences, Instituto Superior Técnico, Universidade de Lisboa, Av. Rovisco Pais, 1049-001 Lisbon, Portugal; 3 Associate Laboratory i4HB – Institute for Health and Bioeconomy at Instituto Superior Técnico, Universidade de Lisboa, Av. Rovisco Pais, 1049-001 Lisbon, Portugal; 4 Instituto de Telecomunicações (IT), Av. Rovisco Pais 1, Torre Norte Piso 10, 1049-001 Lisbon, Portugal; 5 Collaborative Laboratory to Foster Translation and Drug Discovery, 3030-197 Accelbio, Cantanhede Portugal

**Keywords:** Pluripotent stem cells, Stem-cell differentiation, Data mining, Machine learning

## Abstract

The main objective of the present work was to highlight differences and similarities in gene expression patterns between different pluripotent stem cell cardiac differentiation protocols, using a workflow based on unsupervised machine learning algorithms to analyse the transcriptome of cells cultured as a 2D monolayer or as 3D aggregates. This unsupervised approach effectively allowed to portray the transcriptomic changes that occurred throughout the differentiation processes, with a visual representation of the entire transcriptome. The results allowed to corroborate previously reported data and also to unveil new gene expression patterns. In particular, it was possible to identify a correlation between low cardiomyocyte differentiation efficiencies and the early expression of a set of non-mesodermal genes, which can be further explored as predictive markers of differentiation efficiency. The workflow here developed can also be applied to analyse other stem cell differentiation transcriptomic datasets, envisaging future clinical implementation of cellular therapies.

## Introduction

Cardiovascular diseases are, according to the World Health Organization, the leading cause of death in the world, representing 32% of all deaths in 2019^1^. Although the heart is composed of a variety of cell types, its functional units are the cardiomyocytes which perform a coordinate contraction, ultimately allowing the blood to be pumped for the entire body. When myocardial tissue is damaged, as in the case of myocardial infarction, there is substantial cardiomyocyte (CM) death. As adult CMs are unable to proliferate^2^, the damaged tissue is replaced by fibroblasts that will form scar tissue and impair the normal contractility of the heart. Additionally, unlike in other organs, there is no strong evidence that a pool of CM progenitor cells, capable of replacing the lost CMs, exists in the heart^3^ making the regenerative capacity of this organ residual.

In this scenario, human Pluripotent Stem Cells (hPSCs) are a promising tool to generate human CMs for cell replacement therapies. hPSCs not only have the capacity to extensively self-renew but also can hypothetically be differentiated into any cell type. Several protocols were already reported to successfully promote differentiation of hPSC into CMs^4^. However, there are still major challenges limiting the widespread application of these cells, such as the upscaling of the human induced Pluripotent Stem Cell (hiPSC) differentiation process, the lack of reproducibility, or the still limited understanding of the biological mechanisms involved in hiPSC cardiac differentiation.

In this regard, Omics sciences can bring a wealth of new information that may allow exploring hidden layers of the mechanisms behind the differentiation process. In fact, over the years, the study of the human cell’s transcriptome has allowed a significantly better understanding of the mechanisms behind the cellular processes^5^, different tissue gene expression^6^, and characterization of disease mechanisms^7^. Transcriptomics analysis rely on techniques such as RNA sequencing (RNA-seq), which normally generate information from tens of thousands of expressed genes that cannot be analysed one by one. The high dimensionality of this type of data requires appropriate bioinformatics tools and mathematical algorithms to uncover its whole potential. In particular, there is the need to develop new methodologies to analyse large datasets and to readily portray differences and similarities in gene expression patterns between a high number of samples without the need for an *a priori* selection of data. Unsupervised machine learning methods allow the extraction of patterns from large data sets with the potential of unveiling information that could hardly be obtained with other methodologies. In fact, unsupervised machine learning has been applied to group genes according to their expression pattern since the beginning of microarray experiments. The three most used methods to cluster gene expression data are Hierarchical Clustering (HC), K-means and Self-Organizing Map (SOM), by this order^8,9^. Clustering allows the division of a large set of genes into smaller groups and then to infer biologically relevant information such as co-expression and co-regulation patterns, or even the functional role of unknown genes^8^.

The primary objective of this work was to use a simple unsupervised workflow to analyse and compare, from a whole transcriptome point of view, RNA-seq data from stem cells undergoing cardiac differentiation. The combination of SOM and K-Means algorithms had previously been shown to be an effective strategy to portray differences and similarities in gene expression patterns^10^ and, as such, these algorithms were here used to analyze, in an unsupervised way, the differentiation processes. In a recent work by Branco and colleagues^11^ a new strategy for hiPSCs differentiation into CMs as 3D aggregates was proposed. A comparative transcriptomic analysis, using RNA-seq, was performed between this new method and the standard 2D protocol^12^, revealing that the 3D hiPSC expansion and subsequent cardiac differentiation contributed to a faster cardiac commitment and an earlier CM structural and functional maturation. Throughout these experiments, approximately 1700 genes were found to be significantly expressed. However, the original transcriptomics analysis focused on a selected subset of 249 cardiac-related genes. Although these genes are probably the most relevant for the analysis performed, there is still much unexplored information in this dataset that can deepen our understanding of the cardiac differentiation process. The workflow here described was therefore applied to this RNA-seq dataset to further extend the analysis previously made^11^. The method allowed to visualize the gene expression dynamics throughout the cardiac differentiation process and to identify differences between the 2D and 3D protocols. Remarkably, our analysis allowed to correlate lower differentiation efficiencies with the early expression of a set of genes mostly associated with endoderm differentiation.

## Results

### Methodology overview and dataset description

**Figure 1:**
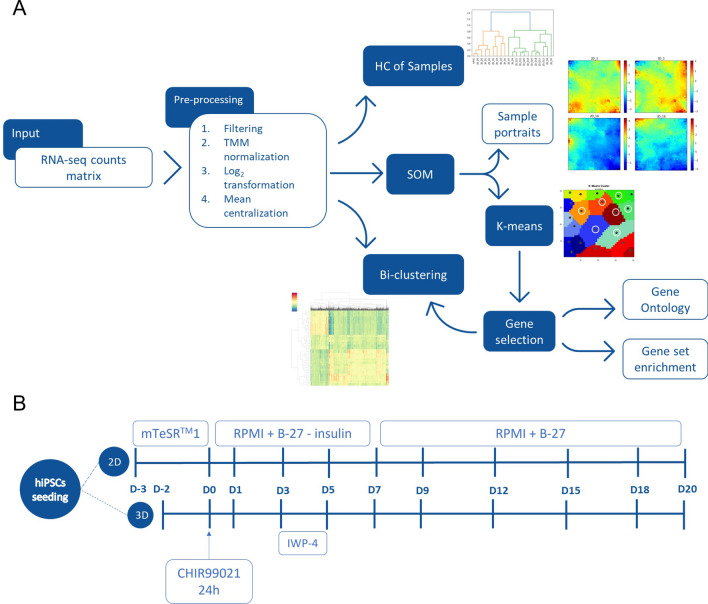
Methodology overview and dataset description **(A)** Overview of the proposed workflow, **(B**) Overview of the hiPSC cardiac differentiation protocols and the time points analysed by RNA-seq in the Branco et al.^11^ data set.

A workflow was designed to combine multiple unsupervised machine learning methods for the analysis of the whole transcriptome of samples obtained during hiPSC cardiac differentiation experiments (Fig. 1A). The final goal was to visualize expression patterns of the complete gene set and to identify, in an unbiased way, the most relevant genes for the analysis of the differentiation process. The complete description of the methodology can be found in the detailed methods section.

After pre-processing the raw data, HC was used on the samples’ dimension. Hierarchical Agglomerative Clustering algorithms organize data into a hierarchical structure, known as a dendrogram. In the beginning, each gene is considered a cluster and, progressively, the algorithms join the most similar clusters until a single cluster, containing all the genes, is obtained. Distinct clustering methods are available, based on the dissimilarity measure between samples and on the definition of dissimilarity between clusters (eg. single-link, average-link or complete-link)^13^. A data partition is obtained by cutting the dendrogram at a specific threshold. In this workflow 9 combinations of metrics (Euclidean, Cosine and Pearson correlation-based distance) and methods (single-link, average-link and complete-link) were tested.

In parallel, SOM, a model-based algorithm, was applied to the genes to portray gene expression landscapes for every sample. SOM starts by randomly choosing a set of $k$ centroids which are linked in a grid structure and are called nodes^14^. At each iteration, one gene is selected and assigned to the most similar node. This node, and its neighbours, will be updated to become more similar to the input gene. As the number of iterations increases, the neighbourhood decreases, and eventually, the nodes become stable. This process of node and neighbourhood updating will cause near nodes in the grid structure to be more similar than those in far positions of the grid. At this point, all genes are mapped to the most similar node and all nodes are, for the last time, updated to the average of all genes mapped to it forming an entity here called metagene. This final step is what allows for the creation of a topology representation of the input genes’ expression patterns and directly comparing different samples. Among the SOM implementations used to study transcriptomic data, the OposSOM package^15^ has been widely used and has proved to be a versatile tool in different contexts^16–18^ and therefore was the one selected.

The next step consisted of using the K-means algorithm for gene selection and to form biologically relevant gene clusters through the differentiation and between culture methods. The K-means algorithm is a partitional clustering method that subdivides the genes into a predefined number of $k$ clusters^13^. Starting with $k$ centroids randomly chosen from the gene set, the algorithm iterates between a partitioning step, which consists in assigning genes to the cluster corresponding to the nearest centroid, and a centroid update step, which is the computation of the average of the genes in each cluster, until a stable partition is obtained.

After this step, other biological analyses were made, such as Gene Set Enrichment analysis and Gene Ontology over-representation. Lastly, Hierarchical Bi-Clustering (HBC) was performed simultaneously on the genes and samples’ dimensions. HBC is a method based on HC which is widely used for gene expression data. This method simultaneously performs clustering over the samples and the genes, providing a final representation, usually as a heatmap with the samples’ and genes’ dendrograms on the top and side, allowing the visualisation of gene expression patterns for different samples.

As previously mentioned, the RNA-seq data from Branco et al.^11^, which is available through Gene Expression Omnibus^19^ (GEO) Accession Number GSE116574, was reanalysed in this work. This data set is composed of samples from cardiac differentiation using the temporal modulation of the Wnt signalling pathway under a standard 2D condition and as 3D aggregates. Briefly, after a short hiPSC expansion phase (2–3 days), differentiation is induced at day 0 through the activation of the Wnt signalling pathway, with the small molecule CHIR99021. The cells were kept in an RPMI medium supplemented with B-27 without insulin. From day 3 to day 5 the Wnt pathway was inhibited by supplementing the medium with the IWP-4 small molecule. From day 7 onwards the culture medium was changed to RPMI medium with insulin.

For both conditions, samples were collected at ten different time points, as represented in Fig. 1B. Each condition has 3 replicates (except day 12 of the 2D differentiation that was performed in duplicate) which together with the hiPSCs seeding samples from which each both 2D and 3D differentiation started, accounts for a total of 62 samples.

### Hierarchical agglomerative clustering to establish a whole transcriptome sample hierarchy, replicate variability and information loss

In the previous work by Branco et al.^11^, differential expression analysis between the 2D and 3D differentiation protocols and culture days was made using a set of 249 cardiac-related genes. Considering that the relationship between samples, based on the entire transcriptome, is not yet known and that differential expression analysis of such high number of genes is too laborious, hierarchical clustering (HC) was used to organize and distinguish groups of similar samples as well as to establish the overall hierarchy created with all the samples.

**Figure 2:**
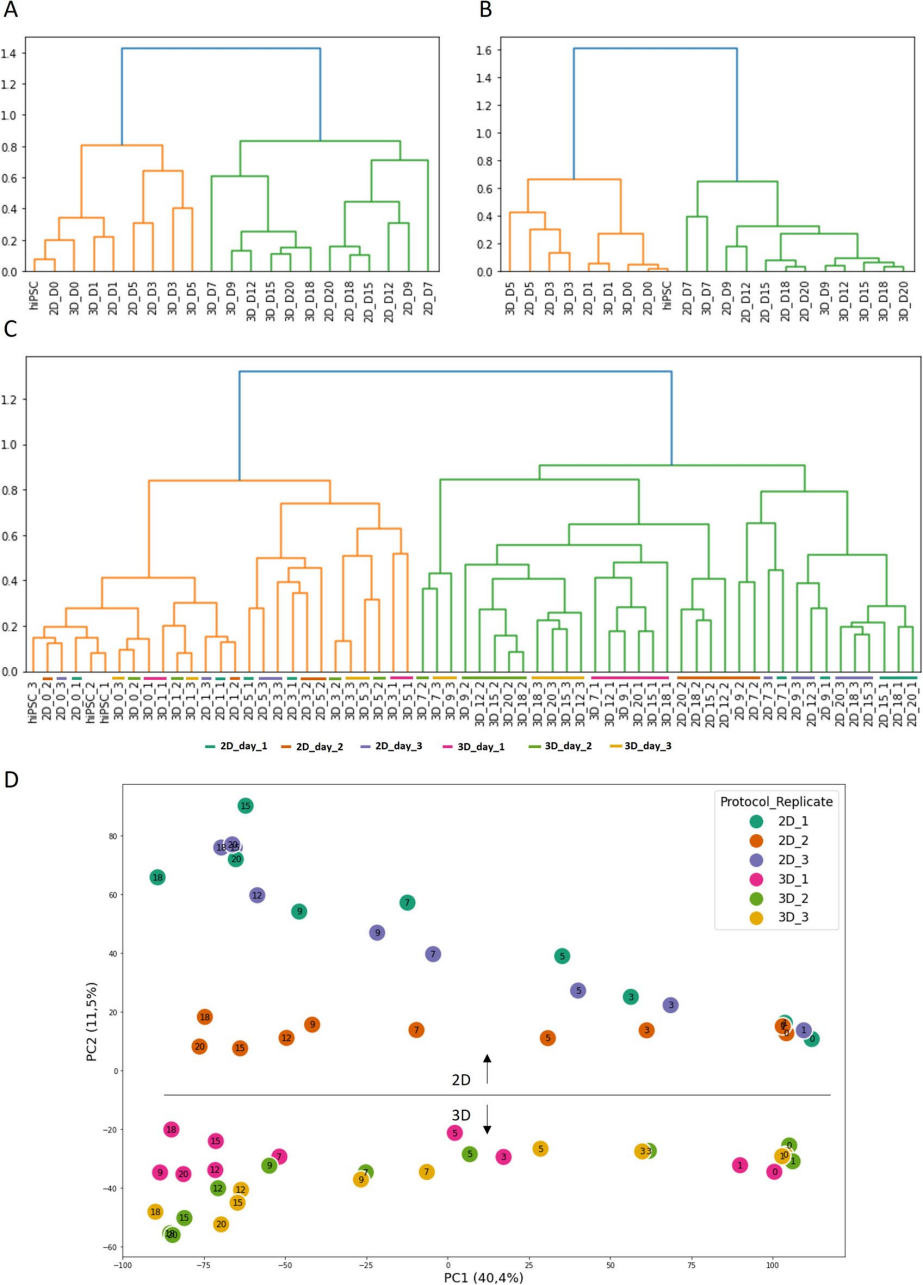
HC to establish a whole transcriptome sample hierarchy, replicate variability and information loss **(A, B)** Dendrograms considering the average of the replicates for each time point, **(A)** considering the whole geneset and **(B)** considering the subset of cardiac-related geneset selected by Branco et al.^11^. Samples are labelled according to [Differentiation protocol_Day]. (**C**) Dendrogram considering each sample and the whole geneset. Samples are labelled according to [Differentiation protocol_Day_Replicate] and marked with colours according to the protocol and replicate **(D)** PC1vs PC2 with all samples from day 0 to 20 and coloured according to the protocol and replicate (colour codes: turquoise—2D replicate 1, orange—2D replicate 2, purple—2D replicate 3, pink—3D replicate 1, green—3D replicate 2, yellow—3D replicate 3).

We started by using HC to generate dendrograms considering the average of the replicates for each time point and the whole gene set, as shown in Fig. 2A. The dendrogram reveals a clear separation of data into two main clusters, one containing samples until day 5 (orange cluster) and another gathering samples from day 7 (green cluster).

By analysing the dendrogram in more detail it was possible to see that the hiPSCs sample is more similar to the 2D day 0 sample than to 3D day 0, which was already noted before^11^, and that day 1 samples are quite similar between culture conditions. Days 3 and 5 group first according to the differentiation protocol (2D or 3D) and then cluster with the previous samples (days 0 and 1). The green cluster is organized temporally, but with complete segregation between 2D and 3D samples. This segregation between 2D and 3D samples is an indication that the culture formats are diverging in gene expression patterns. For both differentiation protocols, there is a cluster shift on day 7 possibly associated with IWP4 supplementation, which promoted cardiac specification and, consequently, a major change in gene expression patterns. Moreover, day 7 samples are isolated from the rest of the cluster which can also be hypothesised to happen due to the change to an insulin-containing medium afterwards.

The same HC methodology was followed considering only the subset of cardiac-related genes selected in the previous work by Branco et al.^11^. As shown in Fig. 2B, two clusters were also generated, divided by day 7, but, in contrast to the results obtained with the whole gene set, in the cluster with the early samples (orange), days 3 and 5 are not arranged according to the differentiation protocol but temporarily, which is an indication that considering the subset of genes here used, the samples from the same day are more similar to each other regardless of the culture format. Similarly, in the green cluster, day 7 samples are grouped which did not occur considering the entire gene set. Moreover, the remaining 3D samples form a subcluster, just then merged with the 2D samples. This hierarchy may suggest that 3D samples are at a later stage of the differentiation, as far as the cardiac-related genes are concerned. These differences confirm that there is information loss when considering such a small amount of genes.

In the previous examples, the average of the time point replicates was used to generate the dendrograms. However, since variability between replicates is commonly observed in hiPSC differentiation experiments, a dendrogram was generated with each replicate individually plotted (Fig. 2C). Despite the increased visual complexity of the dendrogram, two clusters divided by day 7 are obtained, increasing evidence that between days 5 and 7 major shifts in the transcriptome are seen and the impact of the Wnt signalling pathway inhibition can be advanced as the major driver for the transcriptional changes occurring during this differentiation.

Analysing the orange cluster, it is possible to observe that the replicate samples from the initial time points are similar, but from day 3 onwards, different time points and protocols are interspersed, probably representing the gradual increase in variability as the differentiation progresses. On the green cluster, there is still a separation between 2D and 3D samples but not directly into two sub-clusters, as in Fig. 2A, being no longer possible to create clusters with all samples from each differentiation method. With a closer look, it is possible to notice that some samples start to be grouped not according to the differentiation stage, but by the replicate, which is unexpected and suggests that different gene expression patterns may be observed in the replicates.

To complement the dendrogram results, a PCA analysis was also performed (Fig. 2D). By analysing PC1 vs. PC2 it is possible to understand that the samples are displayed in a temporal fashion according to PC1 and divided between 2D and 3D protocols by PC2. There is some grouping by replicate (example 2D replicate 2 marked in orange), but it is not possible to retrieve the grouping by replicate observed in the dendrogram. Overall, the first 2 principal components do not allow to infer the hierarchy observed with hierarchical clustering, most probably because just around 50% of all variation is observed with these components while the dendrogram C represents the calculated distances between each sample considering all genes.

### Self-organising map portraits reveal different gene expression patterns throughout differentiation and between protocols

**Figure 3:**
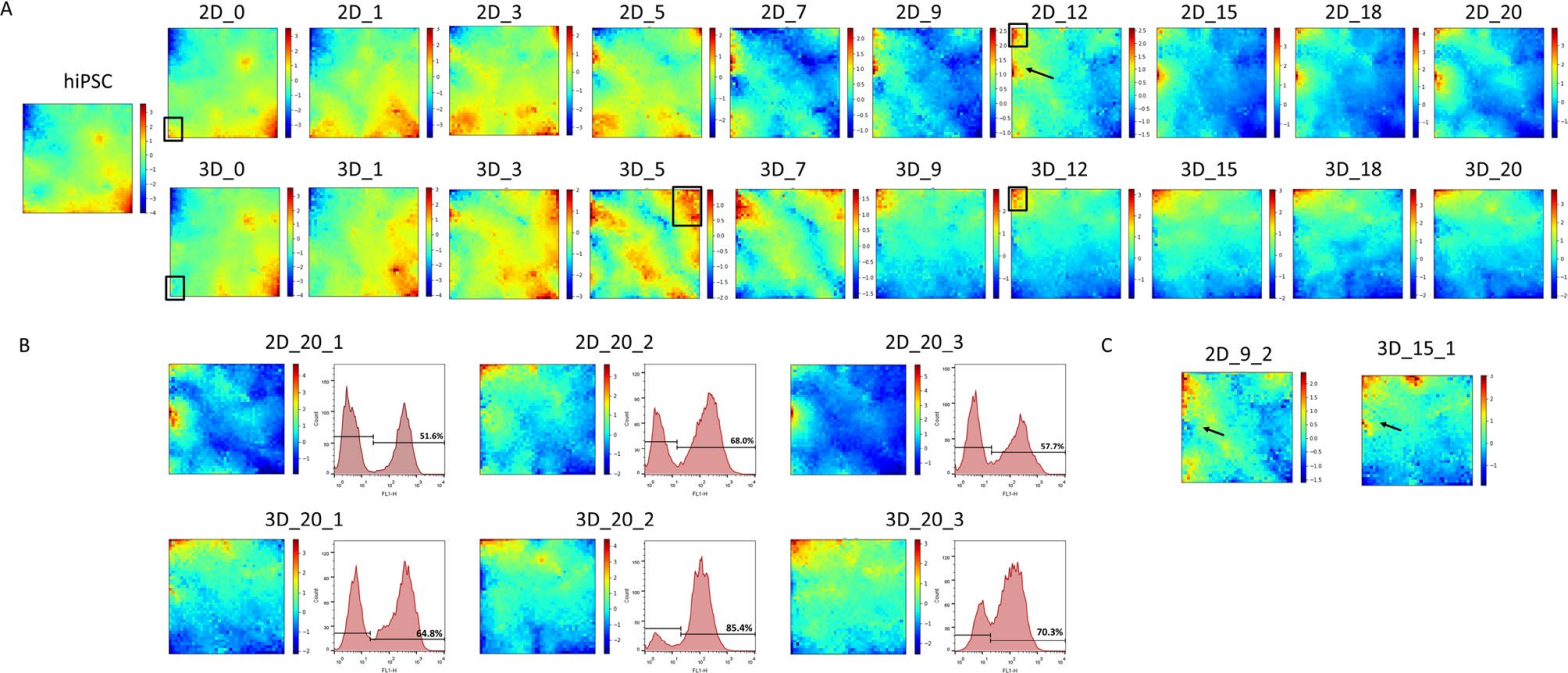
SOM portraits reveal different gene expression patterns throughout differentiation and between protocols **(A)** SOM portraits of the average of the replicates for each time point, for both 2D and 3D cardiac differentiation protocols; **(B)** Day 20 portraits for each replicate and final percentage of cTNT^+^ cells, as determined by flow cytometry analysis; **(C)** Portraits for samples 2D_9_1 and 3D_15_1 with a black arrow highlighting the metagenes associated with the final cardiomyocyte content. Portraits are labelled according to [Differentiation protocol_Day] and [Differentiation protocol_Day_Replicate], respectively, and colour bars represent $log_{2}FC$ values of expression.

Hierarchical clustering on the dimension of the sample can provide information on the relationship between samples and even replicates. However, this approach does not identify which genes are responsible for such structure and does not extract biological information regarding the genes involved in the differentiation process. To analyse in more detail the changes in the transcriptome and to visualise this high dimensional data, a SOM algorithm was used. As performed in the HC analysis, initially, SOM portraits of the average of the time point replicates were plotted (Fig. 3A) and then individual sample portraits were created (supplementary figure 1). All SOM portraits are plotted on a relative scale in which for each sample, dark blue and red represent the lowest and highest expression values, respectively, in log Fold Change (logFC).

From the analysis of the average SOM portraits (Fig. 3A), it was possible to confirm the high similarity between the hiPSCs seeding and day 0 samples. However, this data representation highlights the differences between 2D_D0 and 3D_D0 samples, visually attributed to a group of metagenes in the lower-left corner of the portrait (black rectangle on days 0 portraits), which are not expressed on the 3D day 0 sample. Surprisingly, the portraits for samples from days 1 until 5, which form the first cluster observed previously (Fig. 2A), are considerably different between culture formats. The major difference between these samples consists of the lack of expression of the genes in the lower-left corner of the grid (black rectangle) during the 3D protocol. Furthermore, the 3D protocol displays a higher expression of the genes in the upper right corner of the grid (black rectangle on portrait 3D_5) when compared with the 2D one. Overall these portraits raise the hypothesis that the culture format strongly influences the patterns of gene expression during the initial days of differentiation and the impact of such variation may be further studied. In both differentiation protocols, the transcriptomes converge, in the final time points studied, to similar states with an area of over-expression in the upper-left corner of the portraits (black rectangle on days 12 portraits). However, an over-expressed spot in the middle of the left edge of the 2D differentiation portraits (black arrow on portrait 2D_12) is also observed. This subset of genes represents a major difference between the end-stage of the two protocols. In the 2D protocol, the expression of this subset of genes starts around day 9. Although the 2D portraits seem more similar to the 3D ones at this stage, this may be causing the complete segregation between protocols later observed in the HC dendrograms (Fig. 2A, green cluster).

In order to better understand the variability among the individual time point replicates, SOM portraits for each replicate were generated (supplementary figure 1). It is possible to observe that until day 1 all replicates are essentially identical to each other, but, from day 3 onwards, much more variability is noticeable, as previously described for the dendrogram representation (Fig. 2C). In particular, on day 9 in the 2D protocol, the second replicate does not present the over-expression of the metagenes at the middle of the left edge of the grid (highlighted with a black arrow Fig. 3C). Moreover, for the subsequent days, this area is never over-expressed in opposition to the increase in expression for the other replicates. As for the 3D replicates, on day 15 of replicate 1, the same previously mentioned spot is expressed but with less intensity (highlighted with a black arrow on Fig. 3C).

To better understand the differences between replicates of the same protocol, the final percentage of cTNT^+^ cells for each replicate, which is a measure of the final efficiency of CM differentiation, was also considered (Fig. 3B). It is possible to notice that the replicates with lower differentiation efficiency are the ones with higher expression of the metagenes previously highlighted with a black arrow on the left edge of the portrait (Fig. 3A). Also, the second replicate of the 2D differentiation, which does not show expression of this group of metagenes, had a comparable differentiation efficiency, for instance to the first replicate from the 3D differentiation. However, due to the low number of samples, it cannot be stated that the expression of these genes is correlated with the lower efficiency of the process.

### Self-organising map portraits accurately display commonly known genes for different stages of hipsc cardiac differentiation

**Figure 4:**
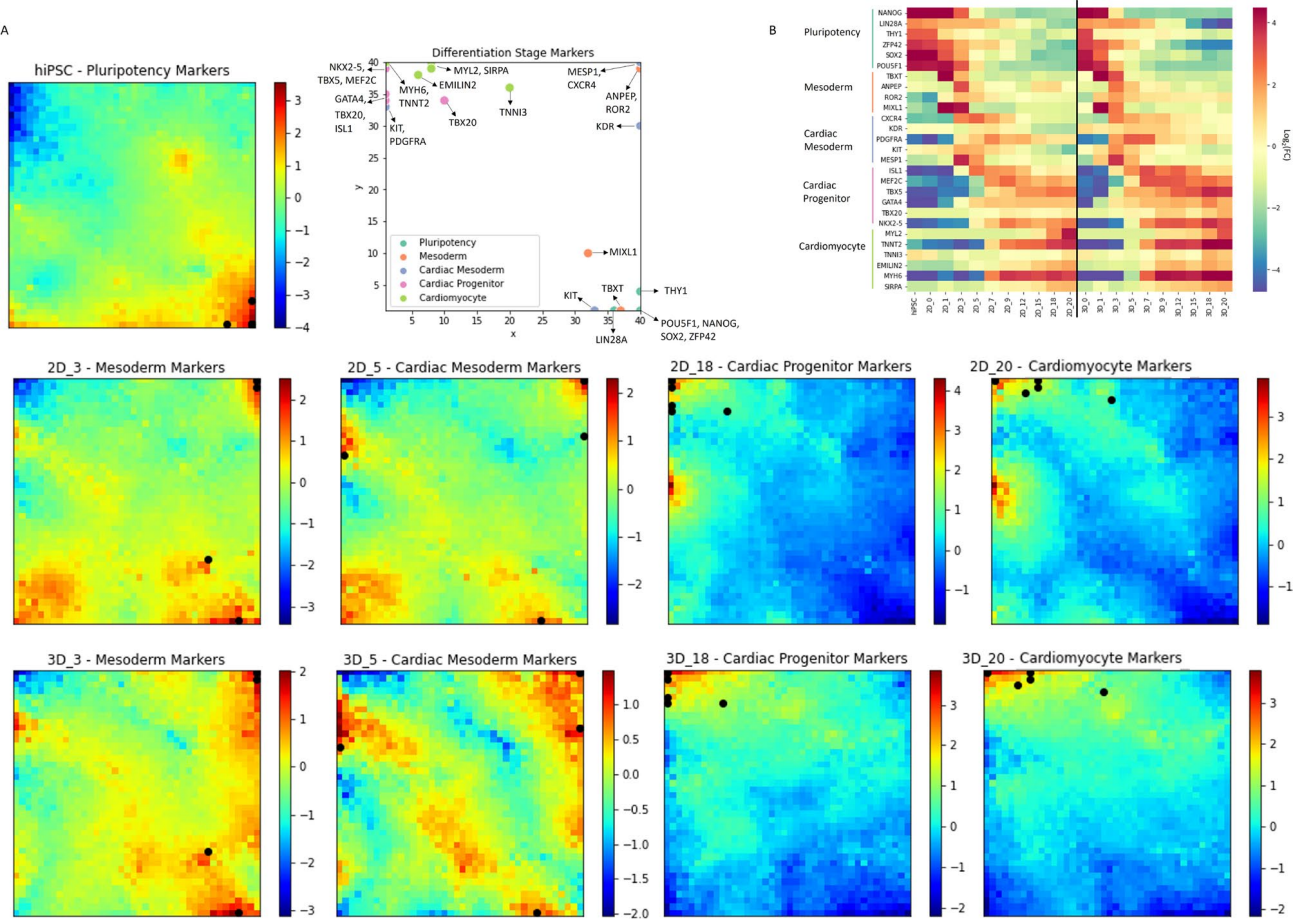
Location of common differentiation stage markers in the SOM grid and overlap with average portraits from hiPSC, days 3, 5, 18 and 20; **(A)** **Pluripotency (turquoise)**—POU5F1 (40x1), NANOG (40x1), SOX2 (40x1), LIN28A (36x1), ZFP42 (40x1), THY1 (40x4) **Mesoderm (orange)**—TBXT (37x1), ANPEP (40x40), MIXL1 (32x10), ROR2 (40x39) **Cardiac Mesoderm (purple)**—MESP1 (40x40), KDR (40x30), KIT (33x1), CXCR4 (40x40), PDGFRA (1x33) **Cardiac Progenitor (pink)**—ISL1 (1x34), NKX2-5 (1x40), GATA4 (1x35), TBX5(1x40), TBX20 (10x34), MEF2C (1x39) **Cardiomyocyte (light green)**—MYH6 (1x40), TNNT2 (1x40), TNNI3 (20x36), MYL2 (8x40), EMILIN2 (6x38), SIRPA (8x39). Portraits are labelled according to [Differentiation protocol_Day] and colour bars represent $log_{2}FC$ values of expression; **(B)** Heatmap of stage markers expression (log2FC).

To validate the SOM portraits, the location of commonly known genes that play a crucial role at different stages of hiPSC cardiac differentiation^2^ were plotted onto the SOM grid (Fig. 4). The Pluripotency marker genes, such as Oct4, Sox2 or Nanog, are located in the area of maximum expression for the samples containing cells in the pluripotent state (hiPSCs, 2D day 0 and 3D day 0).

Regarding the Mesoderm markers, it is interesting to see that some are located in the upper right corner of the grid and others in the lower part, near the “pluripotency area”. The distribution of the Mesoderm markers between the areas of over-expression for day 3 reveals that, although these genes are characteristic of the mesoderm stage, their expression profile is substantially different. TBXT is located in the area of over-expression shared with the pluripotency samples, indicating that the expression of this gene is rapidly decreasing afterwards, and may have started even before day 3. ROR2, which is located in the upper right corner of the grid on the contrary is not expressed before day 3 and sustains its expression for longer periods of time. This pattern was previously experimentally identified for a similar differentiation protocol^20^ and is a good indicator that the SOM is indeed able to accurately represent the subtle differences in expression patterns. Additionally, genes located in the same node and/or area of the grid could be further tested as alternative marker genes since they have the same expression pattern throughout the experiment. As for the Cardiac Mesoderm markers, they are located on three different edges of the grid, coincident with the over-expressed metagenes for day 5.

Likewise, the Cardiac Progenitor and Cardiomyocyte markers were also found in the area of over-expression for the final samples for both culture formats. However, the markers of CMs are spread in the area just expressed in the 3D samples at the end of the differentiation, corroborating the previous analysis by Branco *et al.*^11^ which reveals a higher degree of maturity of the 3D-generated CMs.

### Biological interpretation and single gene expression dynamics

**Figure 5:**
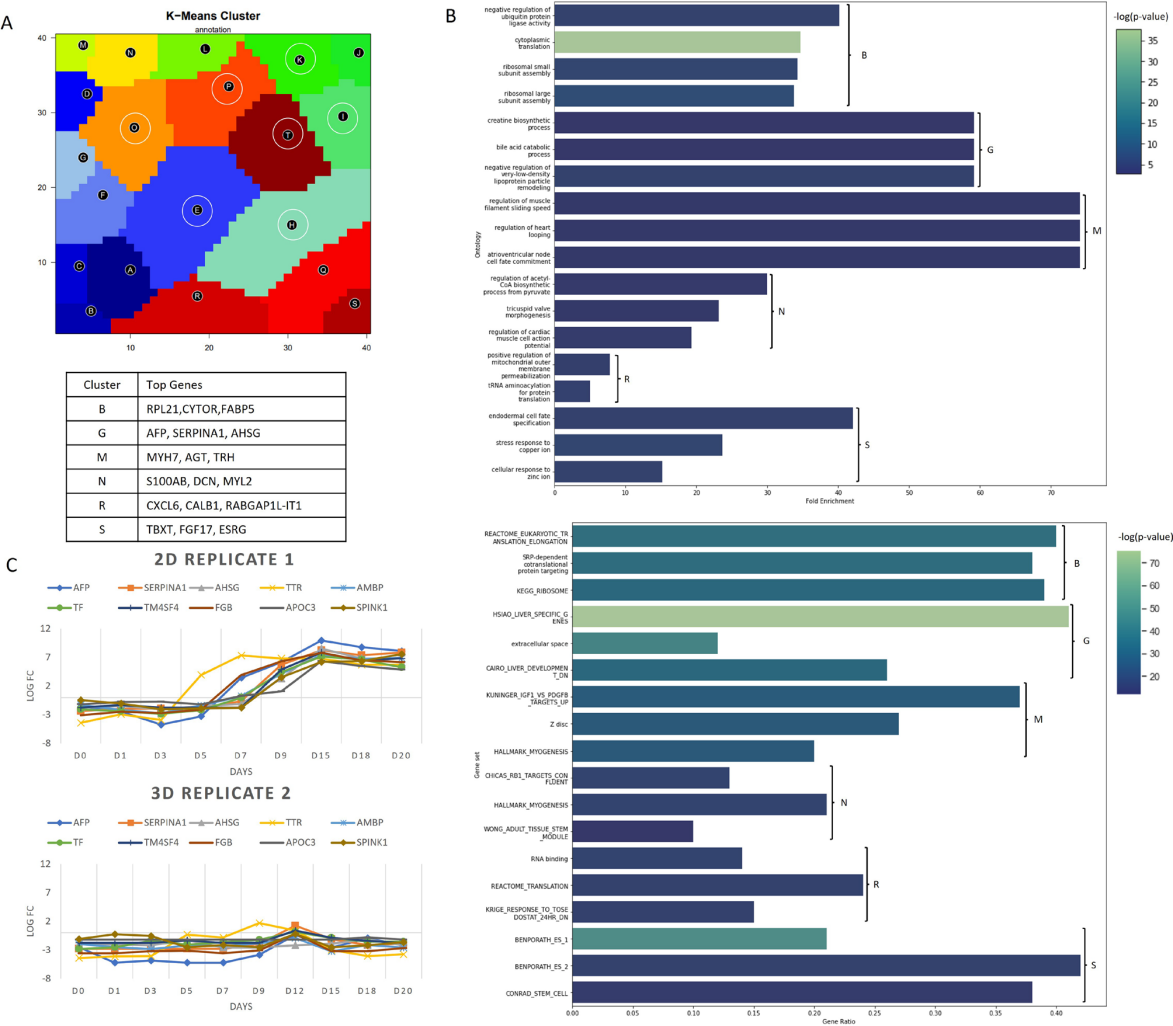
Analysis of the K-means clusters on the SOM grid, **(A)** Representation of the clusters with white circles representing clusters removed from further analysis and top expressed genes for the most relevant clusters (B, G, M, N, R and S); **(B)** top Gene Ontology overrepresentation and Gene set Enrichment for these clusters; **(C)** Expression of the 10 proposed efficiency marker genes for replicate experiments 2D replicate 1 and 3D replicate 2, the lowest and highest in cardiomyocyte content at the final day, respectively.

To study in more detail the over-expressed areas of the grid, the K-means algorithm was used to divide the metagenes into clusters. The resulting partition is presented in Fig. 5A where clusters considered to have a non-significant expression in any sample (low variability over time) are marked with white circles. HC dendrograms were recomputed to assess whether the genes contained in these clusters are sufficient to reconstruct the hierarchy observed using the whole geneset (Fig. 2A). In fact, as shown in supplementary figure 2 using the average of the replicates for each time point, it was possible to obtain the same clusters as the ones previously obtained for the whole geneset.

Gene Set Enrichment (GSE) analysis and Gene Ontology (GO) over-representation were done for the 13 significantly expressed clusters and a complete set of results can be found in supplementary tables 1 and 2 and a summary of the most relevant findings is plotted in Fig. 5B.

Clusters M and N, which are over-expressed at the end of the differentiation, presented an over-representation of genes from several ontologies related to cardiac development as well as an enrichment in gene sets as the hallmarks of myogenesis.

Cluster S, which is the main cluster expressed in pluripotent hiPSC samples, is enriched in gene sets over-expressed in stem cells, however, from the GO no such clear over-representation was seen. Clusters B and R, which are expressed in hiPSCs samples and at the beginning of the 2D differentiation but not in the 3D protocol, are correlated with genes regulating the translation process and ribosomal assembly. It was recently proposed that both the translational process and ribosome biogenesis and homeostasis are key elements in the regulation of stem cell characteristics^21^. The fact that the day 0 samples from the 3D protocol do not express this cluster also suggests that the 3D culture format is priming the cells to a more committed state.

For cluster G, which corresponds to the area of over-expression in the replicates which yielded a lower percentage of cTNT^+^ cells, it was found two genesets with genes over-expressed in the liver and also GO correlated with liver and kidney functions. These results allow hypothesising that endoderm differentiation may be occurring concurrently, leading to a reduction in CM differentiation efficiency. From the developmental perspective, this result is not surprising as both mesoderm and endoderm are derived from the mesendoderm which, in mammals, is formed by the activation of the Wnt/b-catenin pathway together with Nodal and Activin^22^. Furthermore, recent results from single-cell RNA-seq also revealed the presence of endoderm-like cells arising around day 5 of the differentiation^23,24^.

Since expression of the genes in this cluster was found to be correlated with the final efficiency of CM differentiation, the 10 genes with higher expression values (AFP, SERPINA1, AHSG, TTR, AMBP, TF, TM4SF4, FGB, APOC3, SPINK1) were retrieved and their expression through time plotted for each experiment (Fig. 5C and supplementary figure 3). As these genes were found to have an expression of up to 7 logFC at differentiation day 9, they may be further investigated as markers for early prediction of low cardiac differentiation efficiency.

The SOM portraits generated allowed to have a visualization of differences in the transcriptome throughout hiPSC differentiation using two different protocols. However, using only this representation, it is not straightforward to assign these differences to specific genes. HBC was then used to further investigate and visualize the transcriptomic data. Initially, a bi-cluster heatmap with the entire gene set and all the replicates was performed, as well as with the samples’ average (supplementary figure 4A). However, due to the high number of genes, it was not possible to extract meaningful information from these representations. A simplified bi-cluster heatmap was then generated, using only the genes from the K-means clusters previously considered relevant (supplementary figure 4B). Using this subset of genes, different expression patterns became more evident and it was possible to clearly identify a set of genes which are highly expressed only in the 2D differentiation samples, replicates 1 and 3, from day 9 to 20.

Nonetheless, this representation is still difficult to interpret and therefore we decided to further investigate the SOM results by generating a heatmap (supplementary figure 5A) considering only the SOM clusters G, M, N and S, which have the higher expression values and their biological significance already clarified. Also, to facilitate the interpretation, in supplementary figure 5 the samples were not clustered. Using this simplified heatmap, a 5-cluster cut was done, providing a cluster where the highly expressed genes for 2D replicates 1 and 3 can be isolated. Supplementary figure 5B presents a magnification of this cluster and, remarkably, 9 out of the 10 genes previously proposed as potential early markers of differentiation efficiency are here depicted and marked in light green. With this comparative analysis between SOM and HBC, it can be concluded that in contrast to SOM, HBC is not suitable for a whole-genome representation, but the use of both can be complementary since they provide visualizations with different resolutions of the gene expression patterns. While SOM portrays global gene expression patterns, in the form of metagenes, HBC can be used to generate heatmaps that allow the visualisation of single gene expression dynamics.

## Discussion

Cardiovascular diseases are a huge burden on healthcare systems worldwide and have profound impacts on society^25^, being the focus of intense research to meet the undeniable need for better and more effective treatments. Cellular therapies are evolving and may become promising alternatives to the current therapeutic programs but there are still important challenges to address before a clinically approved treatment can be made available.

hPSCs are the perfect raw material for the *in vitro* mass production of CMs, which can be used in multiple therapeutic approaches. Despite the recent advances in the field, hPSC cardiac differentiation still presents some limitations, including the lack of efficient and scalable protocols for CM generation. During the differentiation process, hPSCs are expected to undergo significant gene expression changes in response to the differentiation stimuli and a study of this dynamic transcriptome is expected to provide a wealth of new information that ultimately will improve the CM production, both quantitatively and qualitatively. However, such a whole transcriptome study was not yet presented. The in-depth analysis of the increasingly high number of available hPSCs cardiac differentiation transcriptomic datasets requires an easy-to-use and robust workflow.

In this work, a whole transcriptome analysis methodology which combines different unsupervised machine learning approaches is applied to extend the previous analysis by Branco *et. al*^11^ to study the transcriptional changes between two different hiPSC cardiac differentiation protocols.

HC was used for a sample-centred analysis, to reveal the overall hierarchy of the samples, demonstrating the impact of Wnt signalling pathway inhibition at the transcriptional level. This algorithm also allowed us to observe the impact of selecting a restricted number of genes which causes information loss and can be visually identified by a disruption in the overall relationship between samples. Furthermore, the similarity between replicates could be evaluated by analyzing the organisation obtained in the dendrogram C.

SOM provided a whole transcriptome visualization of each sample highlighting the differences and similarities in transcriptional states between differentiation time points and protocols. With this new visualization of the transcriptome, we confirmed that heterogeneity between replicate samples is present. We were also able to observe that, regardless of the culture platform used (2D or 3D), a subset of genes was overexpressed in samples with low cardiac differentiation efficiency (low % of cTNT positive cells). It was also interesting to observe that the “3D-day 0” samples do not express a group of metagenes present in the hiPSC samples and in the “2D-day 0” samples. Moreover, despite the final convergence of both protocols to the same highly expressed metagenes, between days 3 and 7 the areas of high expression differ between protocols, suggesting different gene expression “trajectories” throughout the differentiation process. Overall, the methodology here presented allowed to obtain information that would not be uncovered without the analysis of the whole transcriptome and would not be detectable with, for instance, the PCA analysis shown in Fig. 2D.

The K-means algorithm was applied on the SOM nodes to create 20 clusters of metagenes and clusters with significant gene expression over time were selected for further analysis. Using these clusters, we were able to confirm previously described results in an unsupervised way and also to find new and unexpected gene expression patterns. This analysis revealed that endoderm-related genes were over-expressed in the replicates with low CM differentiation efficiency. As a complementary approach, Hierarchical Bi-cluster was used to visualize the transcriptome, remarkably confirming the correlation between the expression of the identified set of genes and the efficiency of the process. Another interesting result retrieved from this clustering result was the understanding that the group of genes that ceased to be expressed in the 3D culture conditions right from day 0 (Clusters B and R) are annotated as regulators of the mRNA translation process and ribosomal assembly. The expression of these genes and the processes which they regulate have been associated with undifferentiated stem cells and therefore it would be interesting to further understand the impact of these genes in the differentiation process.

Typically, the progression and efficiency of hiPSC differentiation into CM is monitored for instance by counting the number of beating aggregates or using the gold standard flow cytometry analysis of specific sarcomeric and surface stage-related markers^3^. Despite the quantitative and qualitative information obtained with this method, there is a lack of monitoring strategies for the early detection of low differentiation efficiency outcomes, which is of particular importance in large-scale cultures for future industrial cell manufacturing. Having these considerations in mind, the 10 genes with higher expression (supplementary figure 3) were identified as good candidates to predict the CM differentiation efficiency at an early stage. The expression of this group of genes was found as early as differentiation day 7-9 and thus the possibility to predict deviations at this early stage could be addressed in more detail in future studies. Since RNA-seq is still considerably expensive and also not suitable for daily monitoring, simpler techniques such as real-time quantitative PCR or flow cytometry analysis could be used for this purpose. The function and biological relevance of such genes is also an important aspect to further take into account. Likewise, the role and impact of the genes found on clusters B and R should be further studied as this knowledge may be important to better understand stem cell commitment into the cardiac and other differentiated lineages.

Unlike the commonly used approaches that focus the transcriptomics analysis on a subset of genes considered to be differentially expressed or relevant for the aims of the study, this holistic and unsupervised analysis and visualization of the complete set of transcripts present throughout the differentiation process has the potential to unveil new relevant information that, by other means, would not be discovered, ultimately improving our understanding of the differentiation processes.

Beyond the new information uncovered in this work, future work opportunities have been identified. While the strategy here presented was informative to highlight differences between replicates within this dataset, it will be necessary to evaluate if these differences are universal across experiments and cell lines for cardiac differentiation. It will be also important to perform the experimental validation of the early low-efficiency marker genes here proposed as well as the single-cell deconvolution^26^ of this data to evaluate if there are indeed cells from endodermal lineage in the samples with lower efficiency. Furthermore, this methodology could be further improved by searching on the SOM grid for long non-coding RNAs and micro RNAs as they have been correlated with the mechanisms of differentiation^27^. Moreover, re-training the SOM grid with additional information on splicing variants could also be an interesting approach, as isoform switching was reported during CM maturation^28^.

This work highlights the important role that the application of machine learning algorithms may have for the future developments of stem cell biotechnology. These techniques can be used for the analysis of large Omics data sets, to obtain new insights on stem biology and also for the development of bioprocesses with multiple applications in regenerative medicine and in the pharmaceutical industry.

## Detailed methods

### RNA-seq data pre-processing

To start this workflow, raw read counts from all samples were considered. To filter the dataset, the function filterByExpr (Filter Genes By Expression Level) of the R package EdgeR^29^ was used. This function removes out genes with less than 10 read counts adjusted as counts per million (CPM) and assures that for a gene to be kept in the dataset, at least *n* samples show a significant expression, being *n* the number of samples in the class with fewer replicates.

The geneset was normalized using the TMM method, by the application of the EdgeR function calcNormFactors. With the normalization factors calculated, raw counts were divided by the corrected library size and log2 transformed using the EdgeR function logCPM with a prior count of 3.

Additionally, gene expression data was centralised so that log-fold changes concerning the ensemble average of each gene were obtained. Eq. 1 detail this procedure where $e$ is the log expression vector for one gene and $\langle * \rangle$ denotes the average.

1 $$\begin{aligned} logFC=\Delta e = e-\langle e \rangle _{all\, samples}\,. \end{aligned}$$

For all steps of this work, when referring to gene expression is the same as $\Delta e$ and logFC values are always compared to the mean expression of the gene over all samples.

Besides studying the individual replicates, the average gene expression for each group of replicate samples was computed.

### Hierarchical agglomerative clustering

As a first approach to reveal the relationship between samples, the function cluster.hierarchy.linkage from the SciPy python package^30^ was used. Complete, average, and single linkages were tested, as well as Euclidean, Cosine, and Pearson correlation distances. To separate the clusters the lifetime criteria, proposed by Fred and Jain^31^, available in the BiosPPy^32^ python package, was used. Dendrograms were plotted using the function cluster.hierarchy.dendrogram from the SciPy python package. Each colour in the dendrogram represents a cluster, and samples in blue are the ones composing a single sample cluster.

For the nine partitions thus obtained, two internal cluster validation measurements were calculated: Silhouette Coefficient and Calinski-Harabasz Index available in the scikit-learn python package^33^. The partition with better-defined clusters, which means, the partition with the densest and most well-separated clusters was chosen for further analysis. In practical terms, one of the partitions with the best Silhouette Coefficient and Calinski-Harabasz Index is chosen. When the two validation measurements are not in coherence a partition which is obtained from different combinations of distance metrics and linkages and that has one of the best indexes is chosen.

The results for the internal cluster validation are in supplementary tables 3 to 5.

### Self-organising map

The R package OposSOM^15^ was chosen for the SOM training and mapping since is one of the most used packages for the application of SOM to transcriptomics data sets.

The OposSOM method receives as input the filtered, normalised, log-transformed and centred gene expression matrix. In the context of this SOM implementation, when training the SOM, the only distance available to calculate the winning node is the Euclidean distance and the grid topology available is a square grid. However, the size of the grid should be set by the user and must contain a number of nodes which is one order of magnitude lower than the original number of genes in the dataset.

In this implementation, the authors named the SOM models of metagenes, since, at the end of the training and after the mapping step, they will represent the mean expression of the genes assigned to that SOM node. Like the grid size, the number of epochs should be defined by the users, and in this work, it was experimentally defined so that the SOM grid has a well-defined area with metagenes with lower entropy and variance, and a high gene-metagene correlation, without the need to wait several hours for the outputs. The entropy, variance and correlation definitions can be found in^34^.

A SOM grid size of 40x40 was chosen and 3 epochs were experimentally set as the best training extension.

#### SOM expression portraits

After training, OposSOM automatically maps the genes onto the trained grid. Each node of the SOM grid, or pixel of the SOM portrait, will have several genes mapped onto it forming a small cluster. The mean of this cluster is computed and corresponds to a metagene. From the metagene grid, individual sample transcriptomic portraits are plotted, as well as the mean portrait formed with the mean expression of the samples from the same class.

These portraits are essentially a topographic map where each pixel is coloured accordingly with the expression value of the metagene for that particular sample. In this work, the portraits are created so that, for each sample, the maximum and minimum values of expression are taken to be the maximum and minimum on the colours of the portrait, as such, the metagenes with lower expression are represented in blue, the ones with higher expression in red, and the intermediate values of expression are represented in shades of green and yellow.

#### SOM grid partition by K-means and gene selection

To further analyze the over-expressed and under-expressed spots identified on the SOM portraits, the K-Means algorithm was used to divide the grid into 20 clusters, named A to T. This functionality is built in the OposSOM package.

Since some parts of the SOM grid are composed of practically invariant metagenes, which do not contribute much to the organisation of the samples, some K-means clusters were excluded from the analysis as they were considered to have no significant expression for any sample.

As a proof of concept that the genes contained in the remaining clusters are the ones responsible for the majority of the differences between samples, and that this methodology serves as a feature selection technique, the hierarchical clustering of the samples was reconstructed and the corresponding dendrograms plotted.

#### Biological information extraction from the k-means clusters

To extract biological information about the genes contained in each of the clusters in the study, Gene Ontology (GO) over-representation and Gene Set Enrichment (GSE) analysis were made.

GO over-representation was done using the PANTHER classification system^35,36^ (17.0 release), with a Fisher test, using Homo Sapiens as a reference list and the GO biological process complete as an annotation set.

GSE analysis was done by the OposSOM package, which automatically identifies, from various sources of gene sets, the ones present in the data studied. In total 6324 gene sets available on the GSEA (Gene Set Enrichment Analysis) website (http://www.gsea-msigdb.org/gsea/msigdb/genesets.jsp), were considered in this analysis.

**Table 1:** Gene set categories description.

Category	Description	#Gene sets
Chr	“Gene sets corresponding to each human chromosome and each cytogenetic band”	24
H	“Hallmark gene sets summarize and represent specific well-defined biological states or processes and display coherent expression”	50
C2	“Gene sets in this collection are curated from various sources, including online pathway databases and the biomedical literature”	4373
BP	“Gene sets derived from the GO Biological Process ontology”	1186
CC	“Gene sets derived from the GO Cellular Component ontology”	326
MF	“Gene sets derived from the GO Molecular Function ontology”	365

The gene sets are subdivided into categories, briefly explained in Table 1, with the description taken from the GSEA website directly.

### Hierarchical bi-clustering

Knowing that SOM can create easy-to-interpret portraits of the sample, hierarchical bi-cluster heatmaps were used as a comparison method.

To perform hierarchical bi-cluster the function clustermap from the Seaborn python package^37^ was used. For this step of the work, the distance metric considered more appropriate was Euclidean distance, as it is the one used by the SOM algorithm, and the linkage providing the most interpretable results was complete linkage.

### Supplementary Information

Supplementary Information.

## Data Availability

The RNA-seq data analysed in this study is available through Gene Expression Omnibus (GEO) Accession Number GSE116574. Until a final version of the workflow is made publicly available, the code used in this study is available from the corresponding author on reasonable request.

## References

[1] WHO. Cardiovascular diseases (CVDs). https://www.who.int/news-room/fact-sheets/detail/cardiovascular-diseases-(cvds). Accessed 16 May 2022 (2021).

[2] Burridge P, Keller G, Gold J, Wu J (2012). Production of de novo cardiomyocytes: Human pluripotent stem cell differentiation and direct reprogramming. Cell Stem Cell.

[3] Kempf H, Andree B, Zweigerdt R (2016). Large-scale production of human pluripotent stem cell derived cardiomyocytes. Adv. Drug Deliv. Rev..

[4] Branco MA, Cabral JM, Diogo MM (2020). From human pluripotent stem cells to 3D cardiac microtissues: progress, applications and challenges. Bioengineering.

[5] Van Verk MC, Hickman R, Pieterse CM, Van Wees SC (2013). RNA-Seq: revelation of the messengers. Trends Plant Sci..

[6] Aguet F (2020). The GTEx consortium atlas of genetic regulatory effects across human tissues. Science.

[7] Jiang W, Chen L (2021). Alternative splicing: Human disease and quantitative analysis from high-throughput sequencing. Comput. Struct. Biotechnol. J..

[8] D’haeseleer P (2005). How does gene expression clustering work?. Nat. Biotechnol..

[9] Oyelade J (2016). Clustering algorithms: Their application to gene expression data. Bioinform. Biol. Insights.

[10] Schmidt M, Loeffler-Wirth H, Binder H (2020). Developmental scRNAseq trajectories in gene-and cell-state space-the flatworm example. Genes.

[11] Branco MA (2019). Transcriptomic analysis of 3D cardiac differentiation of human induced pluripotent stem cells reveals faster cardiomyocyte maturation compared to 2D culture. Sci. Rep..

[12] Lian X (2012). Robust cardiomyocyte differentiation from human pluripotent stem cells via temporal modulation of canonical Wnt signaling. Proc. Natl. Acad. Sci..

[13] Aggarwal C, Reddy C (2018). Data Clustering: Algorithms and Applications. Chapman & Hall/CRC Data Mining and Knowledge Discovery Series.

[14] Kohonen T (2014). MATLAB Implementations and Applications of the Self-Organizing Map.

[15] Löffler-Wirth H, Kalcher M, Binder H (2015). oposSOM: R-package for high-dimensional portraying of genome-wide expression landscapes on bioconductor. Bioinformatics.

[16] Kunz M (2018). RNA-Seq analysis identifies different transcriptomic types and developmental trajectories of primary melanomas. Oncogene.

[17] Schmidt M (2022). Single-cell trajectories of melanoma cell resistance to targeted treatment. Cancer Biol. Med..

[18] Arakelyan A (2021). Transcriptome patterns of BRCA1-and BRCA2-mutated breast and ovarian cancers. Int. J. Mol. Sci..

[19] Barrett T (2012). NCBI GEO: archive for functional genomics data sets-update. Nucleic Acids Res..

[20] Tompkins JD (2016). Mapping human pluripotent-to-cardiomyocyte differentiation: Methylomes, transcriptomes, and exon DNA methylation memories. EBioMedicine.

[21] Gabut M, Bourdelais F, Durand S (2020). Ribosome and translational control in stem cells. Cells.

[22] Vliet PV, Wu SM, Zaffran S, Pucéat M (2012). Early cardiac development: A view from stem cells to embryos. Cardiovasc. Res..

[23] Ruan H (2019). Single-cell reconstruction of differentiation trajectory reveals a critical role of ETS1 in human cardiac lineage commitment. BMC Biol..

[24] Churko JM (2018). Defining human cardiac transcription factor hierarchies using integrated single-cell heterogeneity analysis. Nat. Commun..

[25] Roth GA (2020). Global burden of cardiovascular diseases and risk factors, 1990–2019: Update from the GBD 2019 study. J. Am. Coll. Cardiol..

[26] Newman AM (2019). Determining cell type abundance and expression from bulk tissues with digital cytometry. Nat. Biotechnol..

[27] Iancu CB (2015). Molecular signatures of cardiac stem cells. Rom. J. Morphol. Embryol..

[28] Murphy SA, Chen EZ, Tung L, Boheler KR, Kwon C (2021). Maturing heart muscle cells: Mechanisms and transcriptomic insights. Semin. Cell Dev. Biol..

[29] Robinson MD, McCarthy DJ, Smyth GK (2010). edgeR: A Bioconductor package for differential expression analysis of digital gene expression data. Bioinformatics.

[30] Virtanen P (2020). SciPy 1.0: Fundamental algorithms for scientific computing in python. Nat. Methods.

[31] Fred AL, Jain AK (2005). Combining multiple clusterings using evidence accumulation. IEEE Trans. Pattern Anal. Mach. Intell..

[32] Carreiras, C. *et al.* BioSPPy: Biosignal processing in Python. https://github.com/PIA-Group/BioSPPy/ (2015). Accessed 16 May 2022.

[33] Pedregosa F (2011). Scikit-learn: Machine learning in Python. J. Mach. Learn. Res..

[34] Wirth, H. *Analysis of large-scale molecular biological data using self-organizing maps*. Ph.D. thesis, University of Leipzig (2012).

[35] Mi H (2021). Panther version 16: A revised family classification, tree-based classification tool, enhancer regions and extensive API. Nucleic Acids Res..

[36] Mi H (2019). Protocol update for large-scale genome and gene function analysis with panther classification system (v.14.0). Nat. Protoc..

[37] Waskom ML (2021). seaborn: Statistical data visualization. J. Open Source Softw..

