# Supplementary material for: Unsupervised analysis of whole transcriptome data from human pluripotent stem cells cardiac differentiation
Source: Sci Rep. 2024 Feb 7;14:3110. doi: 10.1038/s41598-024-52970-z (PMC10850331; doi:10.1038/s41598-024-52970-z)
Supplement: Supplementary file 1 — Supplementary Information. [file 41598_2024_52970_MOESM1_ESM.pdf]

Unsupervised Analysis of Whole Transcriptome Data from Human Pluripotent Stem Cells Cardiac Differentiation

Sofia P. Agostinho[1,2,3,4,*], Mariana A. Branco[1,2,3,5], Diogo E. S. Nogueira[1,2,3], Maria Margarida Diogo[1,2,3], Joaquim M.S. Cabral[1,2,3], Ana L. N. Fred[1,4], and Carlos A. V. Rodrigues[1,2,3]

[1]Department of Bioengineering, Instituto Superior Técnico, Universidade de Lisboa, Av. Rovisco Pais, 1049-001 Lisbon, Portugal
[2]iBB —Institute for Bioengineering and Biosciences, Instituto Superior Técnico, Universidade de Lisboa, Av. Rovisco Pais, 1049-001 Lisbon, Portugal
[3]Associate Laboratory i4HB – Institute for Health and Bioeconomy at Instituto Superior Técnico, Universidade de Lisboa, Av. Rovisco Pais, 1049-001 Lisbon, Portugal
[4]Instituto de Telecomunicações (IT), Av. Rovisco Pais 1, Torre Norte Piso 10, 1049-001 Lisbon, Portugal
[5]Collaborative Laboratory to Foster Translation and Drug Discovery, Accelbio, 3030-197 Cantanhede, Portugal
[*]sofia.p.agostinho@tecnico.ulisboa.pt

Supplementary Figures

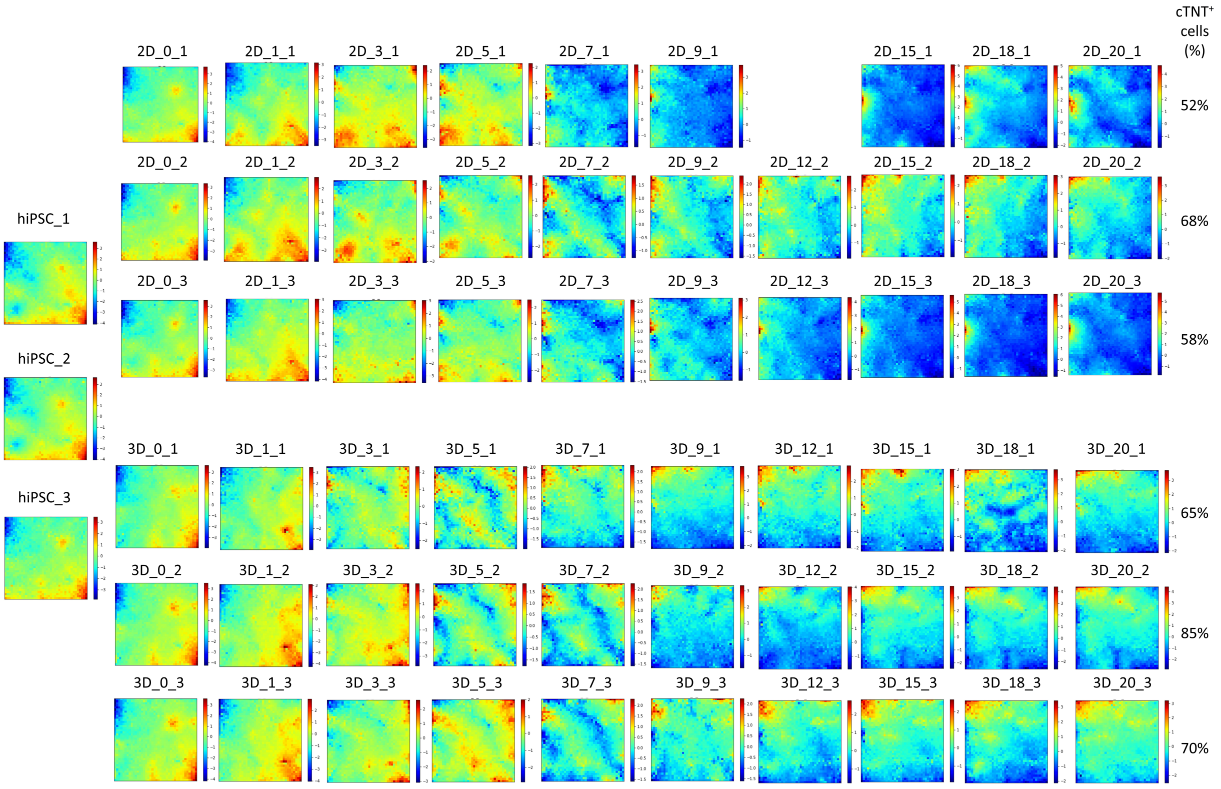

Figure 1. **SOM portraits for each replicate and final percentage of cTNT$^+$ cells, as determined by flow cytometry analysis.** (A) SOM portraits are labelled according to [Differentiation protocol_Day_Replicate] and colour bars represent log_2FC values of expression.

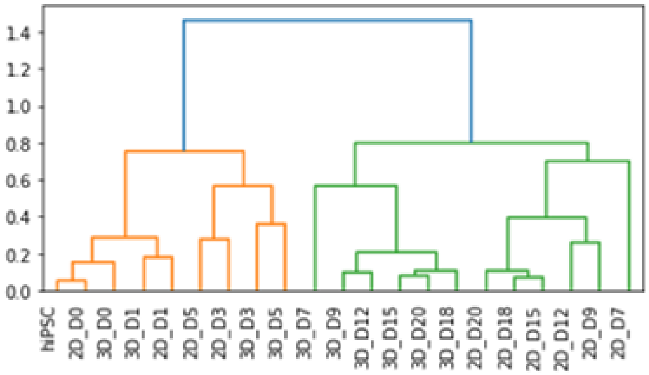

Figure 2. **Gene Selection Information Loss Assessment by Sample Hierarchy Reconstruction** HC dendrogram for the average of the replicates using the genes contained in the 13 significantly expressed K-means clusters. Samples are labelled according to [Differentiation protocol_Day].

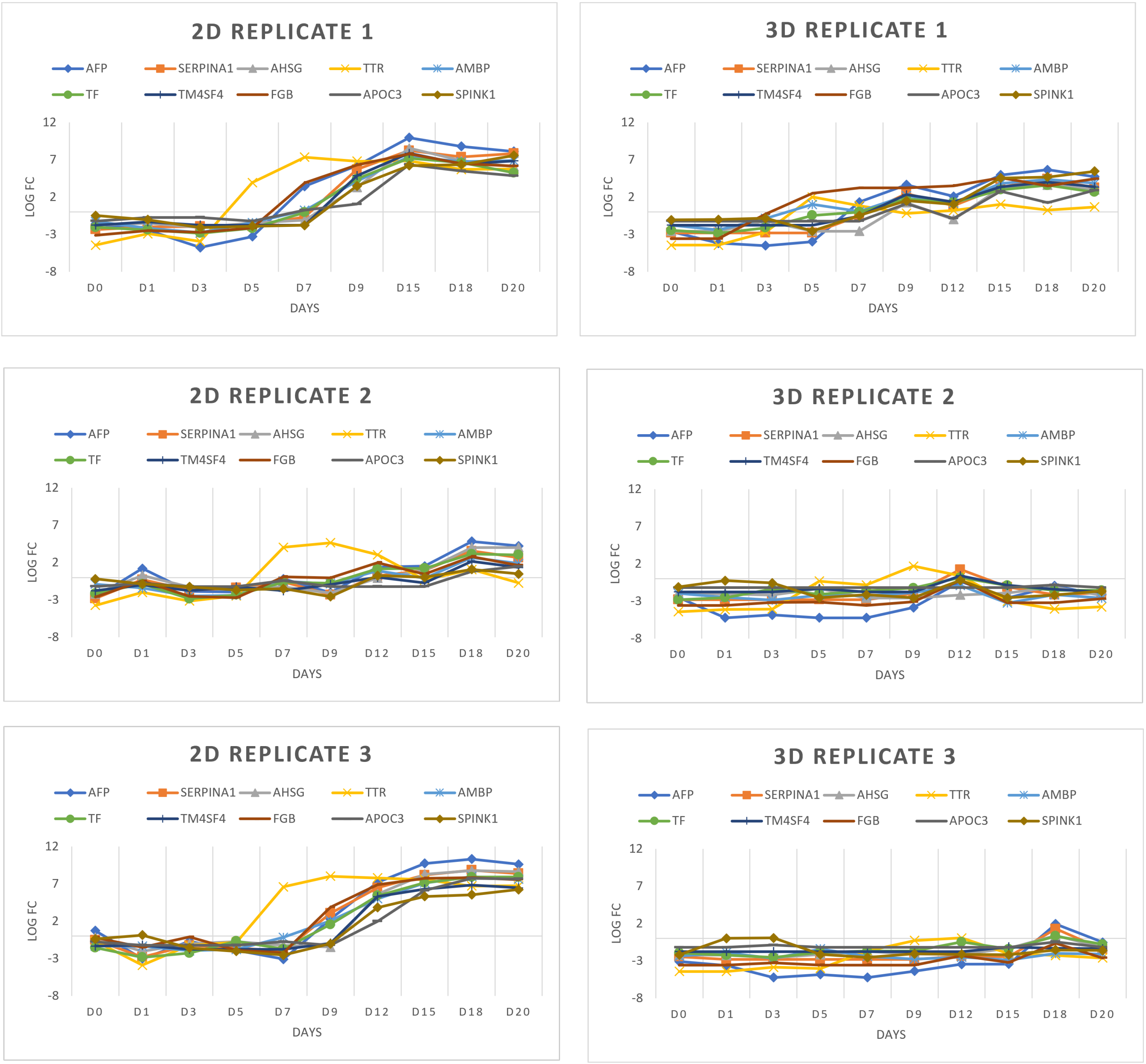

Figure 3. Proposed Markers for Early Prediction of Low Cardiac Differentiation Efficiency Expression, in logFC, of the 10 genes from cluster G with higher expression values (AFP, SERPINA1, AHSG, TTR, AMBP, TF, TM4SF4, FGB, APOC3, SPINK1), throughout time for each differentiation experiment.

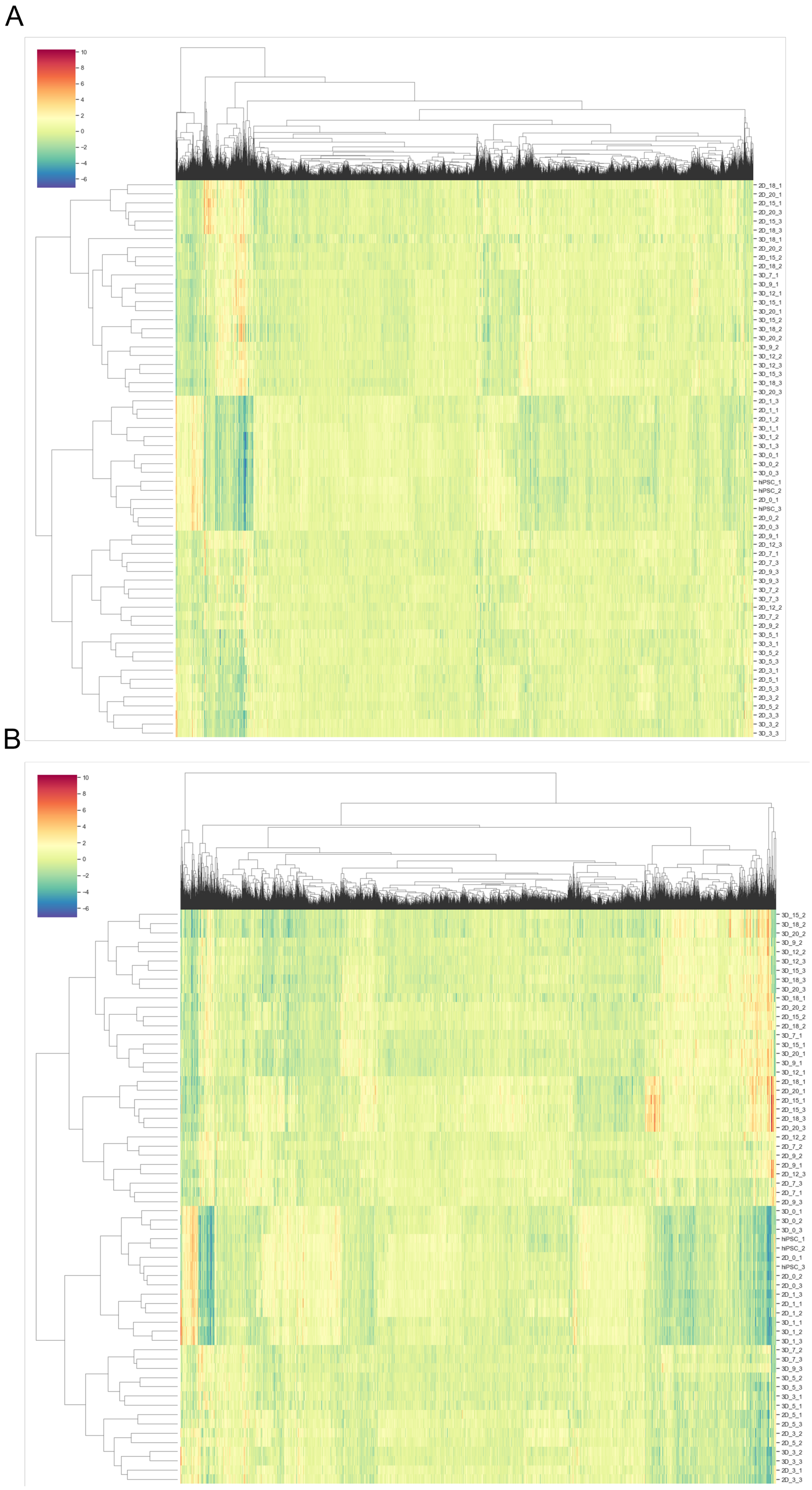

Figure 4. **HBC Provides Single Gene Expression Dynamics Visualization** Hierarchical Bi-clustering with **(A)** the whole set of genes **(B)** the genes contained in the 13 significantly expressed k-means clusters.

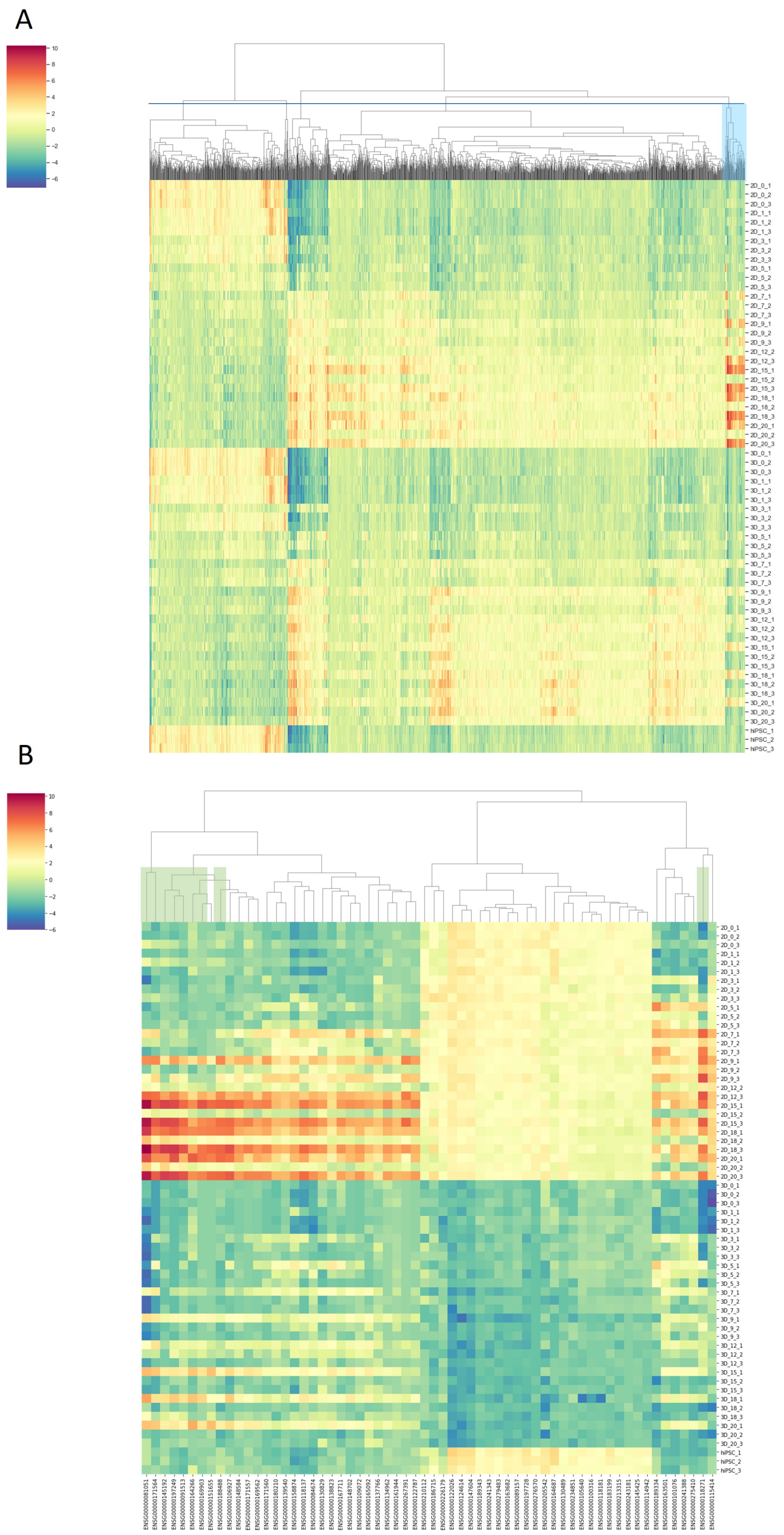

Figure 5. HBC provides single gene expression dynamics visualization **(A)** Hierarchical Bi-clustering with SOM clusters G, M, N and S; **(B)** zoom in the cluster marked in light blue on heatmap A. Light green marked genes are 9 out of the 10 previously proposed for culture monitoring. Samples are labelled according to [Differentiation protocol_Day_Replicate] and colour bars represent log_2FC values of expression.

Supplementary Tables

Table 1. Gene Set Enrichment Analysis Summary Top 3 GSE results for the 13 significantly expressed k-means clusters

Cluster	Gene set	Category	#in/all	p-value
A	Chr16	Chr	48/772	3e-5
	Chr17	Chr	65/1022	1e-3
	Chr22	Chr	27/397	1e-2
B	REACTOME_EUKARYOTIC_TRANSLATION_ELONGATION	GSEA C2	34 / 86	3e-38
	SRP-dependent cotranslational protein targeting	BP	34 / 90	2e-37
	KEGG_RIBOSOME	GSEA C2	31 / 80	1e-34
C	MEISSNER_BRAIN_HCP_WITH_H3K4ME3_AND_H3K27ME3	GSEA C2	37 / 931	6e-05
	ONDER_CDH1_TARGETS_2_DN	GSEA C2	20 / 375	8e-05
	HOLLERN_EMT_BREAST_TUMOR_DN	GSEA C2	9 / 102	2e-04
D	BENPORATH_SUZ12_TARGETS	GSEA C2	89 / 766	6e-42
	BENPORATH_ES_WITH_H3K27ME3	GSEA C2	90 / 790	9e-42
	BENPORATH_EED_TARGETS	GSEA C2	86 / 764	2e-39
F	FEVR_CTNNB1_TARGETS_UP	GSEA C2	66 / 552	3e-13
	CREIGHTON_ENDOCRINE_THERAPY_RESISTANCE_5	GSEA C2	58 / 448	3e-13
	extracellular region	CC	117 / 1382	1e-11
G	HSIAO_LIVER_SPECIFIC_GENES	GSEA C2	75 / 182	2e-76
	extracellular space	CC	107 / 926	9e-48
	CAIRO_LIVER_DEVELOPMENT_DN	GSEA C2	44 / 169	1e-34
J	BENPORATH_SUZ12_TARGETS	GSEA C2	52 / 766	2e-12
	BENPORATH_PRC2_TARGETS	GSEA C2	38 / 446	4e-12
	BENPORATH_EED_TARGETS	GSEA C2	50 / 764	2e-11
L	BENPORATH_ES_WITH_H3K27ME3	GSEA C2	79 / 790	2e-21
	BENPORATH_SUZ12_TARGETS	GSEA C2	68 / 766	9e-16
	BENPORATH_EED_TARGETS	GSEA C2	67 / 764	3e-15
M	KUNINGER_IGF1_VS_PDGFB_TARGETS_UP	GSEA C2	27 / 73	5e-29
	Z disc	CC	31 / 117	4e-28
	HALLMARK_MYOGENESIS	H	36 / 179	7e-28
N	CHICAS_RB1_TARGETS_CONFLUENT	GSEA C2	65 / 519	4e-18
	HALLMARK_MYOGENESIS	H	37 / 179	8e-18
	WONG_ADULT_TISSUE_STEM_MODULE	GSEA C2	60 / 590	1e-12
Q	FISCHER_DREAM_TARGETS	GSEA C2	287 / 921	1e-99
	GOBERT_OLIGODENDROCYTE_DIFFERENTIATION_UP	GSEA C2	193 / 541	4e-85
	DUTERTRE_ESTRADIOL_RESPONSE_24HR_UP	GSEA C2	146 / 312	3e-83
R	RNA binding	MF	197 / 1419	1e-17
	REACTOME_TRANSLATION	GSEA C2	66 / 281	7e-17
	KRIGE_RESPONSE_TO_TOSEDOSTAT_24HR_DN	GSEA C2	142 / 925	2e-16
S	BENPORATH_ES_1	GSEA C2	77 / 368	2e-51
	BENPORATH_ES_2	GSEA C2	16 / 38	8e-17
	CONRAD_STEM_CELL	GSEA C2	14 / 37	4e-14

Table 2. Gene Ontology Over-Representation Analysis Summary Top Gene Ontologies results for the 13 significantly expressed k-means clusters

Cluster	Ontology	Fold Enrichment	p-value
A	DNA repair	2.98	2.24e-5
	regulation of transcription by RNA polymerase II	1.81	2.35e-6
B	negative regulation of ubiquitin protein ligase activity	40.10	7.31e-6
	cytoplasmic translation	34.68	1.51e-38
	ribosomal small subunit assembly	34.29	9.09e-7
	ribosomal large subunit assembly	33.78	5.88e-9
C	-	-	-
D	positive regulation of cell population proliferation	3.43	1.37e-13
	VEGF-activated platelet-derived growth factor receptor signaling pathway	65.78	6.43e-5
	vascular endothelial growth factor signaling pathway	19.19	3.05e-7
	endodermal cell fate determination	65.78	1.31e-3
F	high-density lipoprotein particle remodeling	11.35	5.03e-5
	reverse cholesterol transport	10.09	8.59e-5
	actin filament bundle assembly	5.22	5.7e-8
G	creatine biosynthetic process	59.16	1.62e-3
	bile acid catabolic process	59.16	1.62e-3
	negative regulation of very-low-density lipoprotein particle remodeling	59.16	4.98e-6
J	pH reduction	48.87	1.14e-4
	somite specification	39.09	1.81e-4
	mesodermal cell fate specification	27.92	3.79e-4
L	lobar bronchus development	29.70	4.01e-4
	lung vasculature development	29.70	4.01e-4
M	regulation of muscle filament sliding speed	74.06	1.04e-3
	regulation of heart looping	74.06	1.04e-3
	atrioventricular node cell fate commitment	74.06	1.04e-3
N	regulation of acetyl-CoA biosynthetic process from pyruvate	29.97	5.18e-4
	tricuspid valve morphogenesis	23.18	8.14e-4
	regulation of cardiac muscle cell action potential	19.31	1.69e-4
Q	CENP-A containing chromatin assembly	18.89	2e-6
	spindle assembly involved in female meiosis I	18.89	3.78e-4
R	positive regulation of mitochondrial outer membrane permeabilization	7.75	4.76e-5
	tRNA aminoacylation for protein translation	4.98	2.53e-5
S	endodermal cell fate specification	42.10	1.13e-5
	stress response to copper ion	23.68	8.28e-7
	cellular response to zinc ion	15.16	7.26e-6

HC Partitions Internal Validation

Table 3. Silhouette coefficient and Calinski-Harabasz index for the HC partitions obtained considering the average of the replicates for each time point and considering the whole geneset.

Linkage	Metric	Silhouette Coefficient	Calinski-Harabasz Index
Complete	Euclidean	0.2742	12.5597
Average	Euclidean	0.3596	12.3677
Single	Euclidean	0.3596	12.3677
Complete	Cosine	0.3594	15.7522
Average	Cosine	0.3594	15.7522
Single	Cosine	0.2136	8.1824
Complete	Correlation	0.3594	15.7522
Average	Correlation	0.3150	14.7927
Single	Correlation	0.2136	8.1824

Table 4. Silhouette coefficient and Calinski-Harabasz index for the HC partitions obtained considering the average of the replicates for each time point and the subset of cardiac-related geneset selected by Branco *et al.*.

Linkage	Metric	Silhouette Coefficient	Calinski-Harabasz Index
Complete	Euclidean	0.5136	31.8815
Average	Euclidean	0.5136	31.8815
Single	Euclidean	0.4488	11.1216
Complete	Cosine	0.5136	31.8815
Average	Cosine	0.5136	31.8815
Single	Cosine	0.5136	31.8815
Complete	Correlation	0.5136	31.8815
Average	Correlation	0.5136	31.8815
Single	Correlation	0.5136	31.8815

Table 5. Silhouette coefficient and Calinski-Harabasz index for the HC partitions obtained considering each sample and the whole geneset.

Linkage	Metric	Silhouette Coefficient	Calinski-Harabasz Index
Complete	Euclidean	0.2210	23.7872
Average	Euclidean	0.2658	28.2464
Single	Euclidean	0.1095	1.6173
Complete	Cosine	0.2859	30.7421
Average	Cosine	0.2859	30.7421
Single	Cosine	-0.0471	0.8641
Complete	Correlation	0.1800	21.8700
Average	Correlation	0.2658	28.2464
Single	Correlation	-0.0471	0.8641
